# Cytomegalovirus-induced hemophagocytic lymphohistiocytosis revealing systemic lupus erythematosus: a case report

**DOI:** 10.1097/MS9.0000000000005104

**Published:** 2026-05-06

**Authors:** Shreyas Ellur, Ayesha Mubeen Farooq, Naman Rai, Sameer Kumar Majety, Vineeth Potluri, Gopichand Muppana

**Affiliations:** a Department of Internal Medicine, Gandhi Medical College, Secunderabad, India; b Department of Clinical Medicine, School of Medicine, Xiamen University, Xiamen, P.R China; c Department of Hospital Medicine, Cleveland Clinic Foundation, Cleveland, U.S.A; d Department of Internal Medicine, National Pirigov Memorial Medical University, Vinnytsia, Ukraine

**Keywords:** autoimmune disease, case report, cytomegalovirus infection, hemophagocytic lymphohistiocytosis, immunosuppressive therapy, systemic lupus erythematosus

## Abstract

**Introduction and Importance:**

Hemophagocytic lymphohistiocytosis (HLH) is a rare, life-threatening hyperinflammatory syndrome caused by excessive immune activation. Secondary HLH may arise from infection, malignancy, or autoimmune disease. Cytomegalovirus (CMV) reactivation and systemic lupus erythematosus (SLE) are recognized triggers, but their coexistence as concurrent causes is extremely uncommon. Early recognition is critical, as delayed treatment significantly worsens outcomes.

**Case presentation:**

A 33-year-old woman presented with prolonged fever, oral ulcers, alopecia, arthralgia, and hepatosplenomegaly. Laboratory tests showed pancytopenia, hyperferritinemia (24 500 ng/mL), hypertriglyceridemia, and hypofibrinogenemia. Autoimmune evaluation confirmed new-onset SLE, while polymerase chain reaction verified CMV reactivation. She met six of eight HLH-2004 criteria, and her HScore was 301 (>99% probability), establishing secondary HLH triggered by CMV in the setting of SLE. Treatment included intravenous methylprednisolone followed by tapering oral prednisolone, cyclosporine, mycophenolate mofetil, hydroxychloroquine, and ganciclovir, with supportive antimicrobials and prophylaxis. The patient improved within 2 weeks, with normalization of ferritin and blood counts. Follow-up was weekly for 1 month, then biweekly, and monthly thereafter.

**Clinical discussion:**

This case highlights CMV-associated secondary HLH presenting in the context of newly diagnosed SLE in a young woman. Overlapping infectious and autoimmune features, along with cytopenias, delayed recognition. Her prompt clinical, hematologic, and ferritin response illustrates the benefit of early diagnosis and immunosuppressive plus antiviral treatment.

**Conclusion:**

Concurrent CMV-induced HLH and new-onset SLE are rare but should be considered in patients with unexplained fever and cytopenias. Maintaining a high index of suspicion and initiating prompt, targeted treatment are essential to achieve remission and prevent relapse.

## Introduction

Hemophagocytic syndrome (HPS), more commonly referred to as hemophagocytic lymphohistiocytosis (HLH), is an acute hematologic hyperinflammatory disorder characterized by excessive cytokine release, uncontrolled macrophage activation, and multiorgan dysfunction^[^1,2^]^. It is a rare and life-threatening condition, with an estimated incidence of 1.2 cases per million per year and a mortality rate approaching 47%^[^2^]^.

HIGHLIGHTS

- Cytomegalovirus (CMV) reactivation can precipitate hemophagocytic lymphohistiocytosis (HLH) in systemic lupus erythematosus.
- This report presents a rare case of CMV-triggered HLH, revealing previously undiagnosed lupus.
- Overlapping clinical features of infection and autoimmune flare complicate early diagnosis.
- Combined immunosuppressive and antiviral therapy led to rapid and sustained remission.
- Early recognition and structured follow-up are keys to preventing relapse and improving outcomes.

HLH is broadly divided into primary (familial) and secondary (acquired) forms. The familial type typically presents in childhood and is often linked to genetic syndromes, such as Chediak–Higashi and Griscelli syndrome, which involve inherited immune dysregulation^[^3^]^. In contrast, the secondary form [secondary HLH (sHLH)], also referred to as macrophage activation syndrome (MAS), can be triggered by infection, malignancy, autoimmune disease, or certain medications^[^2,4^]^.

Among infectious causes, Epstein–Barr virus (EBV), herpes simplex virus (HSV), and cytomegalovirus (CMV) are the most frequently implicated pathogens^[^2,4^]^. CMV infection typically remains latent in immunocompetent hosts but may become life-threatening in immunocompromised individuals, particularly those with autoimmune disorders receiving immunosuppressive therapy^[^1,2,5^]^. Additionally, systemic lupus erythematosus (SLE), a multisystem autoimmune disease, is one of the rheumatologic conditions known to precipitate MAS-associated HLH, with a reported prevalence between 0.9% and 4.6%^[^5,6^]^. MAS-associated HLH represents a serious and underdiagnosed complication, with symptoms often mimicking those of a lupus flare, making early recognition and prompt treatment vital for patient survival^[^6,7^]^.

CMV infection and SLE have each been independently associated with HLH; however, their concurrent occurrence is exceedingly rare. Reported cases most often describe CMV infection arising as a complication during the treatment of SLE-associated HLH^[^8^]^. We describe the case of a 33-year-old woman with active CMV infection who was found to have secondary HLH and previously unrecognized SLE. To our knowledge, this represents the first documented case of CMV-triggered HLH occurring concurrently with an initial diagnosis of SLE in an adult woman. This case underscores the importance of early recognition, comprehensive evaluation, and individualized therapy in managing this rare and potentially fatal triad.

This case has been reported in accordance with the TITAN guideline^[^9^]^.

## Case presentation

A 33-year-old woman from a low socioeconomic background presented with a 1-month history of low-grade fever and a 2-week history of painful oral ulcers, non-bloody diarrhea, generalized weakness, patchy alopecia, intermittent arthralgia, dry cough, and exertional dyspnea. She also reported unintentional weight loss of 8 kg over the preceding month but denied photosensitivity, skin rashes, or Raynaud’s phenomenon (Table 1).

**Table 1:** Timeline of clinical events and treatment.

Time period	Event/Investigation	Key findings/Interventions/Outcomes
Month −2 to −1	Onset of symptoms	Low-grade fever, diarrhea, oral ulcers, alopecia, arthralgia, progressive fatigue, 8-kg weight loss.
Day 0 (Admission)	Initial evaluation	Pale, febrile, hepatosplenomegaly, cervical lymphadenopathy; pancytopenia, elevated LDH and transaminases.
Days 1–2	Infectious and autoimmune screening	CMV IgM positive, PCR confirmed reactivation; ANA 1:640 (homogeneous), anti-dsDNA positive, low complement, direct Coombs positive → newly diagnosed SLE.
Days 3–4	HLH-specific work-up	Ferritin 24 500 ng/mL, hypertriglyceridemia, hypofibrinogenemia; bone marrow biopsy showing hemophagocytosis; six of eight HLH-2004 criteria fulfilled; HScore = 301 (>99% probability).
Days 4–14	Inpatient treatment	IV methylprednisolone (1 g × 3 days) → oral prednisolone taper; cyclosporine, mycophenolate mofetil, hydroxychloroquine; IV ganciclovir for CMV; ursodeoxycholic acid; broad supportive care (antibiotics, antifungal and Pneumocystis prophylaxis, PPI).
Days 5–14	Clinical response	Fever resolved by day 5; ferritin ↓ to 8200 → 2800 ng/mL; hemoglobin 10.1 g/dL, WBC 4200/µL, platelets 145 000/µL at discharge.
Discharge (Days 14)	Transition to outpatient care	Stable vitals; discharged on oral prednisolone 40 mg (taper), mycophenolate, hydroxychloroquine, prophylactic medications, calcium/vitamin D.
Months 1–3	Outpatient follow-up	Weekly → biweekly visits; normalization of liver function and inflammatory markers; gradual taper of steroids.
Months 6	Long-term outcome	Complete clinical and biochemical remission; ferritin 180 ng/mL; stable counts; no lupus flares or opportunistic infections; returned to baseline function.

On examination, she appeared pale and chronically ill. Vital signs were stable, except for a low-grade fever. Bilateral, non-tender cervical lymphadenopathy was present, with the largest node measuring 1.5 cm. Multiple shallow ulcers were observed on the buccal mucosa. Abdominal examination revealed hepatosplenomegaly, with the liver palpable 3 cm below the right costal margin and the spleen 4 cm below the left. Respiratory examination showed fine bilateral inspiratory crepitations. Cardiovascular and neurological examinations were unremarkable, and there were no skin rashes, petechiae, or purpura. No family history of autoimmune disease was identified.

Laboratory investigations (Table 2) revealed severe pancytopenia, elevated lactate dehydrogenase (LDH), and raised inflammatory markers. Liver function tests showed elevated transaminases, mild hyperbilirubinemia, and hypoalbuminemia. Infectious screening was negative for HIV, hepatitis B and C, tuberculosis, and bacterial sepsis, supported by low procalcitonin levels. CMV serology was positive for viral reactivation, which was confirmed by polymerase chain reaction (PCR). Autoimmune workup was consistent with SLE, showing a positive anti-nuclear antibody (1:640, homogeneous pattern), elevated anti-dsDNA, hypocomplementemia, and a positive direct Coombs test indicating autoimmune hemolysis.

**Table 2:** Trend of key laboratory parameters during hospitalization and follow-up.

Parameter	Admission	Day 7	Discharge	1 Month	6 Months
Ferritin (ng/mL)	24 500	8 200	2 800	700	180
Hemoglobin (g/dL)	7.6	9.1	10.1	10.8	11.5
White blood cells (×10^3^/µL)	2.1	3.2	4.2	4.9	5.6
Platelets (×10^3^/µL)	52	90	145	210	240
ALT (U/L)	180	120	70	45	35

ALT, alanine amino transferase.

Given the clinical picture, HLH was suspected. HLH-directed testing demonstrated markedly elevated ferritin, hypertriglyceridemia, and hypofibrinogenemia. Bone marrow biopsy revealed activated macrophages engulfing erythrocytes, leukocytes, and platelets, confirming hemophagocytosis. Imaging showed mild bilateral interstitial infiltrates on chest radiography and hepatosplenomegaly on ultrasound. Although natural killer (NK)-cell function and soluble interleukin-2 receptor assays were unavailable, the patient met six of the eight HLH-2004 diagnostic criteria. Her calculated **HScore was 301**, corresponding to more than 99% probability of HLH. The findings established a diagnosis of **secondary HLH triggered by CMV reactivation in the setting of newly diagnosed SLE**.

Treatment was initiated with intravenous methylprednisolone 1 g daily for 3 days, followed by oral prednisolone 1 mg/kg/day on a tapering schedule. Additional immunosuppressive therapy included cyclosporine 3–5 mg/kg/day, mycophenolate mofetil 1 g twice daily, and hydroxychloroquine 200 mg daily. Intravenous ganciclovir was started for CMV reactivation, and ursodeoxycholic acid was prescribed for hepatoprotection. Supportive care consisted of piperacillin–tazobactam, fluconazole prophylaxis, trimethoprim–sulfamethoxazole for *Pneumocystis pneumonia* prophylaxis, and proton pump inhibitors for gastric protection.

During her 14-day hospital stay, the patient showed steady improvement. Fever subsided by day 5, appetite and energy levels improved, transaminases began to normalize, and no new infections occurred. Ferritin decreased from 24 500 ng/mL at baseline to 8200 ng/mL by day 7 and 2800 ng/mL at discharge. Hemoglobin improved to 10.1 g/dL, leukocyte count to 4200/µL, and platelet count to 145 000/µL. She was discharged on oral prednisolone 40 mg daily (with a structured taper), mycophenolate mofetil, hydroxychloroquine, trimethoprim–sulfamethoxazole, calcium, and vitamin D supplementation.

Follow-up was conducted weekly for the first month, twice monthly for the next 2 months, and monthly thereafter. At 6 months, she remained in complete remission, with no recurrence of fever, cytopenias, or constitutional symptoms. Ferritin normalized to 180 ng/mL, blood counts and liver function were stable, and steroids were tapered to 5 mg daily. There were no lupus flares or opportunistic infections, and she had returned to her baseline level of functioning. Early recognition and prompt initiation of therapy were crucial in achieving this favorable outcome.

## Discussion

### Overview

Secondary HLH (sHLH) is a clinicopathological condition that complicates infections, autoimmune diseases, or malignancies^[^10^]^. It commonly presents with high-grade fever, pancytopenia, hepatosplenomegaly, and abnormal liver function tests, accompanied by histopathological evidence of activated macrophages engulfing hematopoietic cells in the bone marrow, liver, spleen, or lymph nodes^[^3,10,11^]^.

Although the diagnosis of HLH can be confirmed through molecular testing or by fulfilling the HLH-2004 criteria, adult presentations often deviate from classic descriptions, leading to diagnostic delays. Treatment generally involves immunosuppressive and cytotoxic agents, with combination regimens shown to achieve better outcomes than corticosteroid monotherapy^[^3,6^]^.

Cytomegalovirus infection and SLE are recognized triggers of secondary HLH, but the coexistence of CMV infection, SLE, and secondary HLH is extremely rare^[^3,6,7^]^. Most published cases describe CMV infection as a complication developing during immunosuppressive therapy for SLE-related HLH, rather than as an initial trigger. The present case demonstrates the reverse sequence, in which CMV reactivation unmasked previously undiagnosed SLE through the development of HLH. This reverse pattern poses a diagnostic challenge, as initial manifestations may mimic severe infection or primary hematologic disease.

The coexistence of **CMV-induced HLH revealing new-onset SLE** is exceptionally rare. HLH occurs in approximately 0.9–4.6% of SLE cases, typically during flares or later in the disease course; presentation as the first manifestation of lupus is highly uncommon^[^5,6^]^. Viral infections are among the most frequent HLH triggers in SLE, with Epstein–Barr virus being the most reported. While CMV is a recognized viral trigger, it accounts for only about 9% of virus-associated HLH in adults^[^4^]^. A large review of 2197 adult HLH cases spanning four decades found CMV-related HLH to be rare, and a 2022 summary by Rolsdorph *et al* identified just 74 published CMV–HLH cases, most involving underlying immunosuppression, commonly inflammatory bowel disease, not SLE^[^2,4^]^. To our knowledge, only one prior case in the literature has reported acute CMV infection unmasking undiagnosed SLE via secondary HLH. This rarity underscores the clinical relevance of our case and highlights the need for heightened clinical suspicion. HLH in SLE confers significantly higher mortality (up to 35%) compared to lupus alone, and in adults, overall HLH mortality approaches 40–50%^[^4^]^. Recognizing atypical triggers and overlapping autoimmune syndromes early is essential for guiding timely, targeted therapy and improving patient outcomes.

### Pathophysiology

HLH is an aberrant hyperinflammatory and hyperferritinemic immune response syndrome driven by dysregulated NK cells and CD8⁺ cytotoxic T lymphocytes^[^3,12^]^. Under normal conditions, these cells release perforin and granzyme, cytolytic enzymes that induce apoptosis of target cells through pore formation in the target cell membrane. In HLH, this mechanism becomes impaired either because of inherited cytotoxic pathway defects or as a result of an overwhelming immune stimulus, such as CMV infection. The inability to eliminate activated immune cells leads to sustained immune activation and persistent inflammation.

The term MAS refers to HLH that arises in the setting of autoimmune diseases, such as SLE. In this context, chronic immune activation leads to abnormal B-cell stimulation, unregulated complement activity, and heightened interferon signaling. When combined with impaired NK and T-cell cytotoxicity, these processes trigger a hyperinflammatory cascade that progresses from chronic immune dysregulation to acute systemic inflammation^[^13–15^]^.

This inflammatory shift results in a cycle of uncontrolled cytokine release. Massive production of interleukin-1β, interleukin-6, interferon-gamma, and tumor necrosis factor-alpha drives pathological macrophage activation and hemophagocytosis. These processes explain the clinical picture of prolonged fever, cytopenias, hepatosplenomegaly, and multiorgan dysfunction observed in HLH^[^13,16^]^.

In the present case, SLE was likely present in a subclinical state, with early manifestations that were nonspecific and, therefore, unrecognized by the patient. The patient subsequently developed a CMV infection, which acted as a significant immunologic trigger. This led to the onset of severe cytopenias, hyperferritinemia, and hepatic dysfunction, closely temporally associated with evidence of CMV infection, making an isolated SLE flare less likely as the primary driver. Instead, the CMV infection appears to have exacerbated the underlying autoimmune dysregulation associated with SLE, leading to catastrophic disease expression and rapid progression to secondary HLH.

### Clinical features

The clinical presentation in our case closely resembled the hallmarks of HLH. This syndrome typically manifests with prolonged fever, profound cytopenias (often pancytopenia), hepatosplenomegaly, and lymphadenopathy, accompanied by laboratory evidence of extreme inflammation such as hyperferritinemia, hypertriglyceridemia, and hypofibrinogenemia^[^17^]^. Our patient presented with persistent fever, severe pancytopenia, marked hyperferritinemia (>24 000 ng/mL), hypertriglyceridemia, and organomegaly, satisfying six of the eight HLH-2004 diagnostic criteria. These clinical features are consistent with secondary HLH^[^17^]^. Additionally, she exhibited signs indicative of an underlying autoimmune process, such as painful oral ulcers, arthralgias, patchy alopecia, Coombs-positive hemolytic anemia, and high-titer antinuclear antibody and anti-double-stranded DNA antibodies, which pointed toward SLE. Autoimmune diseases like SLE are well-recognized precipitants of secondary HLH^[^18^]^, and in such cases, the term MAS is often used to describe HLH arising after a rheumatologic event^[^17^]^. Early recognition of HLH is critical, as it is a life-threatening hyperinflammatory state that can progress to multiorgan failure if not swiftly treated^[^19^]^.

Our case is an extremely rare scenario in which HLH serves as the initial clue to an occult autoimmune disease. Previous reports have described patients with previously undiagnosed SLE presenting acutely with secondary HLH^[^20^]^. This overlap can pose a diagnostic challenge because active lupus flares and HLH share many clinical features, including fever, cytopenias, organomegaly, and liver dysfunction, making differentiation difficult without a high index of suspicion^[^20,21^]^. In our patient, the most likely triggering factor for HLH was reactivation of CMV. Viral infections, especially herpes viruses like Epstein–Barr virus and, less frequently, CMV, are known triggers for HLH in susceptible hosts^[^17^]^. Our patient’s HLH likely resulted from the interaction between her underlying SLE and acute CMV reactivation. Similar cases have been reported where the combination of autoimmune disease and viral infection jointly precipitated HLH^[^17,21^]^. Recognizing this interaction is essential, as both components must be treated simultaneously. In our case, a combined regimen of immunosuppressive therapy for SLE and antiviral treatment for CMV resulted in rapid and sustained clinical improvement. This reiterates that early and aggressive treatment of secondary HLH in the setting of autoimmune disease can be life-saving^[^19^]^.

The detailed clinical course of our patient further reinforces the themes highlighted in the final paragraph and contextualized in Table 3. Similar to several reported cases, HLH emerged as the presenting manifestation of an underlying, previously unrecognized autoimmune disorder, with SLE only becoming evident after comprehensive evaluation. In contrast to cases associated with delayed diagnosis and poor outcomes, the early identification of HLH in our patient, prompted by extreme hyperferritinemia, pancytopenia, and organomegaly, allowed for rapid initiation of targeted therapy. As reflected in Table 3, cases in which viral infection and autoimmune disease coexist, particularly CMV reactivation in the setting of SLE, demonstrate a wide spectrum of outcomes that appear closely linked to the timing of recognition and treatment. Our patient’s favorable clinical trajectory parallels reports in which combined immunosuppressive and antiviral therapy was instituted early and contrasts sharply with fatal cases characterized by advanced disease or diagnostic delay. This comparison further supports the conclusion that prompt recognition of the dual autoimmune and infectious drivers of secondary HLH is critical for achieving remission and preventing mortality.

**Table 3:** Select reported cases of hemophagocytic syndrome in SLE patients or related scenarios, with triggers, treatments, and outcomes. Relapses are noted if reported.

Author (Year)	HLH trigger(s)	Therapy given	Outcome (Follow-up)
Ranabahu M *et al* (2026)^[^22^]^	Acute CMV infection (in an immunocompetent patient; also had histiocytic necrotizing lymphadenitis)	Ganciclovir (antiviral) plus immunosuppressive therapy (corticosteroids; supportive HLH treatment)	**Survived.** HLH resolved; SLE diagnosed during follow-up (no HLH relapse reported).
Alharbi LK *et al* (2025)^[^23^]^	New-onset SLE (previously undiagnosed; no infection found)	High-dose glucocorticoids, intravenous immunoglobulin (IVIG), and Rituximab (anti-CD20) immunotherapy	**Survived.** Rapid improvement within 2 weeks; achieved remission of HLH and control of lupus (no relapse noted).
Peng LY *et al* (2023)^[^6^]^	SLE flare with concurrent infection (pulmonary infection in a known SLE patient)	“HLH-94” protocol immunochemotherapy (Dexamethasone + Etoposide), broad-spectrum antibiotics, IVIG, and plasma exchange support	**Survived.** Made a full recovery; **no relapse** of HLH at 1-year follow-up.
Shao D *et al* (2023)^[^20^]^	Aggressive new-onset SLE (lupus nephritis) **plus** CMV reactivation (latent infection)	**Combination therapy**: high-dose steroids, mycophenolate mofetil and tacrolimus (for SLE/lupus nephritis), Etoposide (HLH), and Ganciclovir (CMV)	**Died.** Developed refractory multi-organ failure despite comprehensive treatment; highlights high mortality when HLH is advanced.
Yasmeen J *et al* (2025)^[^24^]^	SLE flare misidentified as routine disease activity (HLH diagnosis delayed)	Intensive immunosuppression (corticosteroids and other immunosuppressive agents)	**Died.** Patient deteriorated rapidly and succumbed to HLH-related organ failure even with ICU care. Delay in recognizing HLH was a key factor.
So MW *et al* (2014)^[^25^]^	SLE with refractory HLH and autoimmune hemolytic anemia (AIHA)	Escalation to biologic therapy – **Rituximab** – after steroids and cyclosporine failed to fully control HLH/MAS	**Survived.** Rituximab was **successful** in inducing remission of HLH and hemolysis; no further relapses reported in follow-up.

### Diagnosis and differentials

Diagnosing secondary HLH is challenging because its features overlap with other hyperinflammatory conditions such as sepsis, autoimmune flares, and MAS^[^26^]^. The HLH-2004 criteria require at least five of eight findings, including fever, splenomegaly, cytopenias, hypertriglyceridemia, hypofibrinogenemia, hemophagocytosis, reduced NK-cell activity, hyperferritinemia, or elevated soluble IL-2 receptor^[^27^]^. Early recognition is crucial, as timely therapy initiated before progression to a fulminant phase improves survival^[^27^]^.

In adults, HLH is usually secondary and often atypical. Because advanced tests, such as NK-cell function or soluble IL-2 receptor, are not always available, the HScore serves as a practical alternative, using accessible parameters such as fever, hepatosplenomegaly, cytopenias, ferritin, triglycerides, fibrinogen, and bone marrow findings^[^28^]^.

In our patient, both diagnostic frameworks supported the diagnosis. She met more than five HLH-2004 criteria and had an HScore of 301, indicating a greater than 99% probability of HLH^[^28^]^. The combination of persistent fever, hepatosplenomegaly, pancytopenia, hypertriglyceridemia, hypofibrinogenemia, and marked hyperferritinemia confirmed secondary HLH.

The diagnostic process was complex. Initially, fever, cytopenias, and hepatosplenomegaly resembled a viral mononucleosis-like illness, but virological testing confirmed CMV reactivation, a known trigger of HLH^[^2^]^. The coexistence of newly diagnosed SLE further complicated differentiation, as lupus flare or MAS may clinically mimic HLH^[^6^]^. However, very high ferritin (24 500 ng/mL), hypofibrinogenemia, and multilineage cytopenias favored HLH.

Distinguishing HLH from sepsis was also essential. Although the patient presented with high-grade fever, the degree of cytopenias, hepatosplenomegaly, and extremely elevated ferritin– – much higher than typically seen in bacterial sepsis – made sepsis unlikely^[^26^]^. The presence of CMV infection ruled out Epstein–Barr virus and confirmed CMV-triggered HLH in the setting of SLE^[^2^]^.

Therefore, the final diagnosis was sHLH precipitated by CMV infection on a background of SLE, supported by the fulfillment of HLH-2004 criteria, a high HScore, and the systematic exclusion of other close differentials.

The diagnostic process and exclusion of close differentials are further clarified when the clinical timeline and longitudinal laboratory trends are examined, as summarized in Tables 1 and 2. The patient’s subacute constitutional symptoms over 2 months, followed by acute deterioration with pancytopenia, hepatosplenomegaly, and marked hyperinflammation at admission, prompted parallel evaluation for infectious, autoimmune, and hyperinflammatory etiologies. Early serologic and molecular testing confirmed active CMV reactivation, while autoimmune work-up established a new diagnosis of SLE, creating diagnostic uncertainty regarding the dominant trigger of HLH. The markedly elevated ferritin, hypertriglyceridemia, hypofibrinogenemia, and bone marrow hemophagocytosis fulfilled six of eight HLH-2004 criteria, with an HScore of 301 indicating a greater than 99% probability of HLH. Serial laboratory trends demonstrated rapid biochemical and hematologic improvement following initiation of combined antiviral and immunosuppressive therapy, with ferritin declining from 24 500 ng/mL at admission to 2800 ng/mL at discharge and progressive normalization of cytopenias and transaminases during follow-up. Sustained remission at 6 months, without lupus flare or infectious relapse, supports the effectiveness of the chosen management strategy and underscores the importance of integrating dynamic clinical and laboratory data when distinguishing overlapping causes of HLH in complex autoimmune-infectious presentations.

### Treatment

Management of sHLH in adults follows the HLH-94 protocol, which recommends an 8-week course of corticosteroids, with or without etoposide or methotrexate. In infection-associated HLH, immunosuppressive and cytotoxic agents should be used cautiously, and targeted treatment of the underlying cause is essential. For CMV-associated HLH, antiviral therapy with ganciclovir or valganciclovir is first-line, while foscarnet or cidofovir can be used in resistant cases^[^2,3^]^.

In our patient, treatment was initiated to address active SLE, control CMV reactivation, and suppress secondary hyperinflammation consistent with HLH. High-dose intravenous methylprednisolone was administered for 3 days to reduce the ongoing cytokine-driven inflammatory response and organ dysfunction. This was followed by tapering oral prednisolone, along with cyclosporine, mycophenolate mofetil, and hydroxychloroquine. Cyclosporine was introduced as a steroid-sparing immunosuppressive agent to inhibit T-cell activation, which plays a central role in both lupus activity and HLH pathogenesis. Hydroxychloroquine was continued as baseline therapy for lupus control. As CMV infection was identified early, antiviral therapy with ganciclovir was administered to suppress viral replication and ongoing immune stimulation, which could otherwise exacerbate the hyperinflammatory state. Ursodeoxycholic acid was given for hepatic protection, and supportive therapy included piperacillin–tazobactam, fluconazole, trimethoprim–sulfamethoxazole, and proton pump inhibitors. The effectiveness of this therapeutic approach is reflected in the progressive clinical stabilization and laboratory improvement detailed in Tables 1 and 2.

The pathophysiology of HLH involves uncontrolled activation of cytotoxic T cells and macrophages, resulting in excessive interferon-gamma release and stimulation of the JAK–STAT signaling pathway. This pathway represents a therapeutic target, and ruxolitinib has shown potential in attenuating hyperinflammation, though it increases the risk of CMV reactivation, warranting close monitoring^[^3,8^]^. Etoposide, which inhibits T-cell proliferation and cytokine secretion, remains a key therapy but may cause end-organ toxicity in adults with comorbidities; thus, dose adjustments or reduced frequency can be considered^[^3,8^]^.

Findings from Table 3 further support the management approach used in our patient. Reported cases of secondary HLH in the setting of SLE demonstrate considerable heterogeneity in triggers and therapeutic strategies, with outcomes closely linked to early recognition and tailored treatment. Cases involving concurrent viral infection, particularly cytomegalovirus, highlight the importance of combining immunosuppressive therapy with targeted antiviral treatment. In contrast to reports with delayed diagnosis or refractory disease requiring escalation to etoposide-based regimens and associated with high mortality, our patient responded rapidly to corticosteroids, cyclosporine, and antiviral therapy alone. This comparison emphasizes that early, individualized therapy addressing both autoimmune activity and infectious triggers can achieve remission without the immediate need for cytotoxic chemotherapy in selected adult patients.

### Complications

HLH itself represents a severe complication of immune dysregulation, particularly when associated with SLE. It often leads to multi-organ failure involving the lungs, liver, kidneys, heart, and central nervous system. Reported complications include acute respiratory distress syndrome, myocarditis, hepatic failure, renal microangiopathy, and fatal meningoencephalitis^[^3,23,29^]^.

Patients with SLE–HLH are inherently immunocompromised, making opportunistic bacterial, viral, and fungal infections a major clinical concern^[^11,29^]^. Hemodynamic collapse from thrombocytopenia is a common cause of death, along with mortality from the underlying disease or treatment-related toxicities. Some studies have also reported poorer outcomes in male patients compared to females^[^3,30^]^.

In our patient, complications included severe anemia, thrombocytopenia, and hepatic involvement, all of which improved rapidly with corticosteroids and immunomodulatory therapy. No infectious complications occurred during hospitalization, likely due to early diagnosis and vigilant supportive care.

### Prognosis

Historically, HLH carries a poor prognosis, with untreated mortality exceeding 50–80%^[^23,29^]^. Adult patients generally have poorer outcomes than pediatric cases, mainly due to secondary etiology, delayed recognition, multi-organ dysfunction, and treatment-related toxicity^[^11,31^]^. Although biologic agents, such as anakinra (IL-1 blockade), tocilizumab (IL-6 blockade), and ruxolitinib (JAK inhibition), have improved outcomes in refractory disease, relapse remains a concern. Up to one-third of the patients experience HLH reactivation or MAS flares within the first year of remission^[^30,31^]^.

While no definitive prognostic biomarker exists, platelet counts below 40 × 10^9^/L have been linked to poorer survival^[^30^]^. Long-term prognosis depends on adequate control of the underlying SLE, careful corticosteroid tapering, and ongoing immunosuppression^[^11^]^. Close follow-up during the first year is essential, with regular clinical and laboratory monitoring to detect early relapse and guide treatment adjustments.

In our patient, follow-up was conducted weekly for the first month, twice monthly for the next 2 months, and monthly thereafter. At 6 months, she remained in complete remission with normalized ferritin (180 ng/mL), stable blood counts, and normal liver function. Steroids were tapered to 5 mg daily, and there were no lupus flares or opportunistic infections. This favorable outcome underscores the impact of early recognition, timely therapy, and structured follow-up in improving survival in secondary HLH associated with SLE.

When viewed in the context of Table 3, our patient’s outcome aligns with the reported cases demonstrating a favorable prognosis when secondary HLH is recognized early and managed with timely, individualized therapy. In contrast to cases complicated by delayed diagnosis, refractory disease, or advanced multiorgan failure, early control of both autoimmune activity and infectious triggers in our patient likely contributed to sustained remission. This comparison further supports the role of prompt diagnosis and comprehensive management in improving long-term outcomes in adult secondary HLH.

## Conclusion

This case highlights the diagnostic and therapeutic challenges of sHLH in the context of SLE and CMV reactivation. Early recognition, rapid initiation of immunosuppressive and antiviral therapy, and structured follow-up were keys to achieving remission and preventing fatal complications. A high index of suspicion is essential in patients with unexplained fever, cytopenias, and hepatosplenomegaly, particularly in those with underlying autoimmune disease. Multidisciplinary management and close monitoring can significantly improve survival and long-term outcomes in this rare but life-threatening condition.

### *Patient’s Perspective [elicited in patient’s mother tongue and translated to English*]

I was unwell and sick for several months, with constant fatigue and an on-and-off fever, but I never realized that it could be a serious underlying illness. When my condition suddenly deteriorated, I was very much anxious with how quickly everything progressed and how I am currently. I am very much grateful to the doctors for the early identification and swift treatment which has made me fight this illness. I now understand that how fatal this condition could have been. This illness has taught me to never ignore any symptoms and to seek medical care early.

### Limitations

This report describes a single clinical case, and its findings, therefore, lack generalizability. The study is retrospective in nature and based on available clinical data and follow-up records, which may not capture all disease dynamics or long-term outcomes. Additionally, specialized investigations such as NK-cell function and soluble IL-2 receptor assays were unavailable, which could have provided stronger diagnostic confirmation. Despite these limitations, the case offers valuable insight into the early recognition and successful management of CMV-triggered HLH in SLE, particularly in resource-limited settings.

## Data Availability

The data regarding the patient can be submitted by the corresponding author upon reasonable request.
